# Complete restoration of fertility in a patient treated for androgen-secreting granulosa cell tumor - Case report

**DOI:** 10.5935/1518-0557.20230027

**Published:** 2023

**Authors:** Tina Sušanj Šepić, Neda Smiljan Severinski, Senija Eminović, Anđelka Radojčić Badovinac, Alenka Višnić

**Affiliations:** 1 Clinical Hospital Center Rijeka - Clinic for Gynecology and Obstetrics, Rijeka, Croatia

**Keywords:** amenorrhea, granulosa cell tumor, luteinizing hormone

## Abstract

**Case Report:**

A 35-yr-old patient suffering from secondary amenorrhea for two years before she was diagnosed. Secondary amenorrhea occurred after the first normal vaginal delivery, and it was initially associated with breastfeeding and a formerly diagnosed thyroid disease. Transvaginal ultrasound confirmed a tumorous mass of the right ovary. Blood hormone tests detected high serum inhibin B and Anti-Müllerian hormone levels and high androgen level with no signs of virilization. Surgical treatment was indicated for a definitive diagnosis of suspected sex cord-stromal tumor. Right-sided laparoscopic salpingo-oophorectomy was performed, and the histopathological analysis confirmed the diagnosis of granulosa cell tumor adult type. The oncological team recommended adjuvant chemotherapy after the operation, but the patient did not give an informed consent. One month after surgical treatment, spontaneous menstrual bleeding occurred with normalization of sex hormone levels and the menstrual cycle. Nine months after surgical treatment, the patient was examined again due to secondary amenorrhea. Ultrasound confirmed a vital intrauterine pregnancy. The pregnancy course was normal, and the patient had a full-term spontaneous vaginal delivery of her second child.

**Conclusion:**

Restoration of fertility after a temporary loss due to hormone-secreting granulosa cell tumor is possible after sparing surgical treatment. The role of adjuvant chemotherapy is controversial, particularly in patients with stage I-II disease because of the rarity of this tumor and the absence of prospective randomized studies.

## INTRODUCTION

Granulosa cell tumor (GCT) of the ovary is an uncommon neoplasm. Adult-type GCT is the most common sex cord-stromal tumor. These neoplasms account for approximately 2-5% of malignant ovarian malignancies and represent the majority of ovarian tumors with endocrine manifestations. The pathogenesis of tumor development is associated with a mutation of the FOXL2 gene, which participates in the transcription of factors relevant for ovarian development during gonadogenesis (Yang *et al*., 2018). Sex cord-stromal tumors occur in women of all ages, but the frequency increases during the fourth and fifth decade of life. GCT most frequently occurs in middle-aged and menopausal women (Bryk *et al*., 2021). Two non-typical forms of granulosa cell tumor are juvenile and cystic types. Both occur in children and young adults (Harris *et al*., 2020). The juvenile type of this tumor occurs before puberty and causes precocious pseudopuberty.

Menstrual cycle irregularity and secondary amenorrhea are frequent symptoms of women of reproductive age due to tumor-induced hormone production (Blot-Dupin *et al*., 2019). Clinical manifestations result from increased estrogen, Anti-Müllerian hormone (AMH), inhibin B, and, less commonly, androgen production. It is not rare that these tumors are discovered accidentally during regular gynecological examinations without significant symptomatology. A quarter of patients has endocrine manifestations of GCT (Färkkilä *et al*., 2015). Due to estrogen or, less commonly, androgen production, irregular uterine bleeding or secondary amenorrhea is common, and in children, there is also the possibility of precocious puberty (Okawa *et al*., 2020). Acute abdominal symptoms occur due to tumor rupture and hemoperitoneum in 10% of cases, more often than in other ovarian tumors (Sherchan, 2021). Granulosa cell tumors are usually 5 to 15 cm in size and are one-sided in 95% of the cases.

Overall, the prognosis is very good, and the 10-year survival rate is 90%. The malignant potential of the granulosa cell tumor is low, and the most important prognosis factor is the stage of the disease when the treatment is initiated (Khosla *et al*., 2014). Since 90% of tumors are discovered in stage FIGO I (International Federation of Gynecology and Obstetrics), the survival rate is 86-96%. The treatment is surgical with possible chemotherapy in later stages of the disease (Temtanakitpaisan *et al*., 2019).

## CASE PRESENTATION

**Figure 1:**
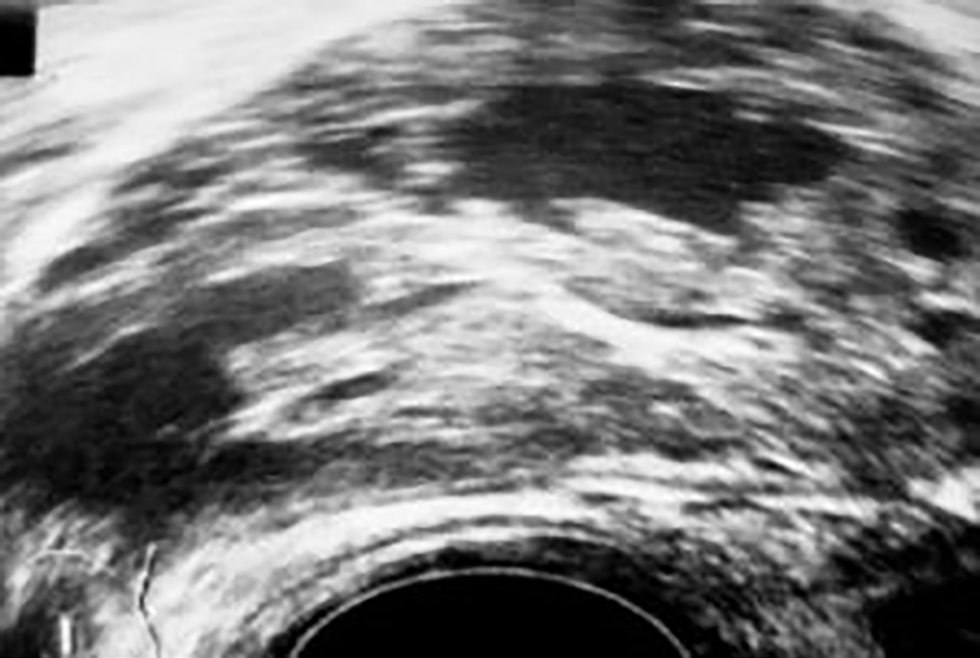
Ultrasonography of the ovarian tumor.

A 35-yr-old patient presented to a gynecological examination due to secondary amenorrhea two years after previous spontaneous pregnancy and normal vaginal delivery. Three years before the first pregnancy, the patient had a thyroidectomy due to suspected thyroid gland malignancy. The histopathological diagnosis revealed follicular adenoma and the patient started with thyroid hormone replacement therapy (levothyroxine^®^ 150µg/day). The menstrual cycle was regular with substitution therapy until the first pregnancy. Amenorrhea after delivery was primarily associated with breastfeeding, but the persistence of amenorrhea even after ceasing breastfeeding and lactation was an unsolved medical condition. The patient was referred to a gynecological endocrinologist. The gynecological examination and transvaginal ultrasound revealed a suspicious, heterogeneous, largely solid, partially cystic tumorous formation of the right ovary, 6 cm in size (Figure 1). Pelvic ultrasonography of the uterus and the left ovary confirmed normal ultrasound morphology, without intraperitoneal free fluid. Serum hormone concentrations were abnormal: elevated luteinizing hormone (LH) levels (21.67 UI/L; normal level in follicular phase 2.4-12.6), and testosterone (2.15nmol/L; normal level 0.29-1.67), decreased follicle-stimulating hormone (FSH) levels (2.42IU/L; normal level in follicular phase 3.3-12.5) and extremely elevated AMH (90.4µg/L; normal level 0.41-6.96) and inhibin B levels (926 ng/L; normal level< 139ng/L). Other hormones, estradiol (87.59pmol/L), prolactin (291mIU/L), as well as tumor markers antigen 125 (Ca 125) (27.1 KU/L), and human epididymis protein 4(HE4) (42.8pmol /L) were within the reference intervals (Table 1).

**Table 1:** Serum hormone values.

Hormone Test	Result	Unit	Ref. Interval	Note
LH (S)	21.67	IU/L	Follicular Phase 2.4 - 12.6 Mid-Cycle 14.0 - 95.6 Luteal Phase 1.0 - 11.4 Postmenopause 7.7 - 58.5	Eclia, Cobas E601 (E411)
FSH (S)	2.42	IU/L	Follicular Phase 3.5 - 12.5 Mid-Cycle 4.7 - 21.5 Luteal Phase 1.7 - 7.7 Postmenopause 25.8 - 134.8	Eclia, Cobas E601 Eclia, Cobas E601
Prolactin (S)	291	mIU/L	Pregnant Women: Dependent on Week of Pregnancy 102 - 496	Eclia, Cobas E601 Eclia, Cobas E601
Estradiol (S)	87.59	pmol/L	Follicular Phase 45 - 854 Ovulation 151 - 1461 Luteal Phase 82 -1251 Postmenopause 18 - 505	Eclia, Cobas E601 Eclia, Cobas E601
Anti-Müllerian Hormone (AAMH) (S)	90.4 H	µg/L	0.41-6.96	Eclia, Cobas E601 (E4u)
Inhibin B (S)	926.0 > H	ng/L	Luteal Phase <92 Follicular Phase <139 Postmenopause <10	Eclia, Cobas E601 (E411)
Testosterone (S)	2.15 H	nmol/L	0.29-1.67	Eclia, Cobas E601 (E411)
Ca 125 (S)	27.1	ku/L	< 35.0	Eclia, Cobas E601
He4 (S)	42.8	pmol/L	< 60.5	Eclia, Cobas E601
Roma Index (Premenopause)	5.5	%	> 11.4 - High Risk for Ovarian Epithelial Cancer < 11.4 - Low Risk for Ovarian Epithelial Cancer	

**Figure 2:**
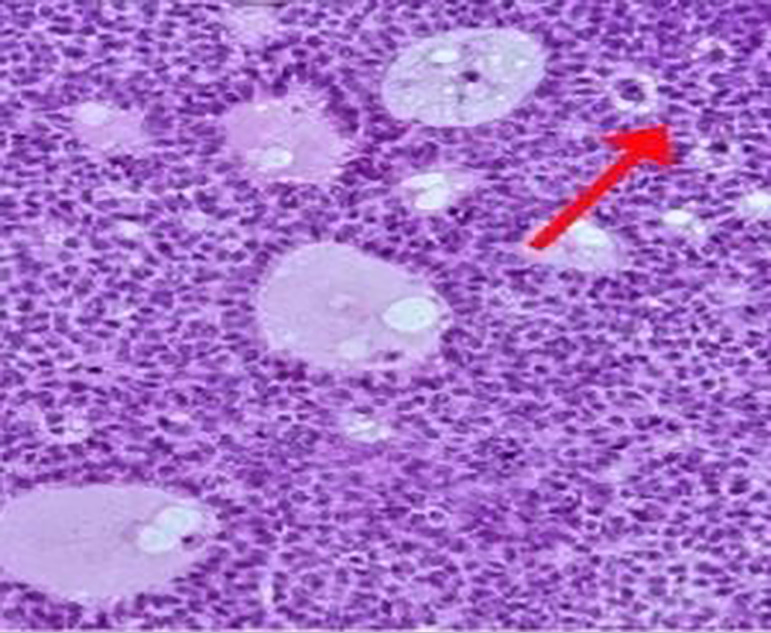
Histologic findings showed granulosa cells with microfollicular growth patterns and Call-Exner bodies (magnified 10 times).

Based on diagnostic tests (ultrasonography and hormones), a sex cord-stromal tumor was suspected, and surgical treatment was indicated. Right-sided laparoscopic salpingo-oophorectomy was performed, the material was sent to intraoperative frozen section consultation, and granulosa cell tumor of the right ovary was confirmed. The tumor ruptured during the surgical treatment and manipulation, which was an adverse event but not rare in granulosa cell tumor surgeries. Biopsy of the left ovary, multiple peritoneum biopsies, and infracolic omentectomy were performed. According to pathologist’s findings, the tumor tissue was macroscopically predominantly solid and yellow. The ovarian tumor tissue was histologically diagnosed as adult-type granulosa cell tumor mostly with diffuse and gyriform growth patterns, to a smaller extent with a microfollicular growth pattern with Call-Exner bodies. Tumor cells were well to moderately differentiated, coffee bean-like in places, with low mitotic activity (Figure 2). Vascular invasion of the tumor was demonstrated by immunohistochemical marker CD34 for vessel endothelium. Lavage and other biopsy materials were cytologically negative, despite local iatrogenic dissemination of tumor tissue during the surgery. The final diagnosis of the disease stage was stage FIGO I C1, and the multidisciplinary team (gynecologist, oncologist, cytologist, pathologist) recommended continuation of chemotherapy, 3 cycles of cisplatin, etoposide, bleomycin (PEB), but the patient disagreed with the recommendation.

One month after the surgical treatment, the patient had her first menstruation. A regular menstrual cycle was established with normal control values of serum sex hormones, and inhibin B levels (61.5ng/L). Nine months after surgery, the patient presented for gynecological examination due to secondary amenorrhea. The ultrasonographic examination confirmed a vital intrauterine pregnancy at 10 weeks of gestation. The pregnancy was normal with levothyroxine substitution therapy 175 mcg/day. In the 39^th^ week of pregnancy, the patient vaginally delivered a healthy male newborn weighing 3900 grams. After the postpartum period, the patient attended regular gynecological follow-ups and showed no signs of relapse 3,5 years after delivery and 5 years after surgery. Three years after surgical treatment, the patient spontaneously conceived third pregnancy, which ended in deliberate termination according to the couple’s decision.

## DISCUSSION

The presented case of a patient with a granulosa cell tumor is interesting because of the androgen activity of the granulosa cell tumor and the reproductive age of the patient at which this tumor is generally less frequently diagnosed (Bús *et al*., 2017). These tumors are most frequently diagnosed after menopause, although they can occur at any age (Madill *et al*., 2020). Relatively young patient age and prolonged amenorrhea after normally conceived pregnancy and childbirth is an unusual clinical presentation of the granulosa cell tumor described in this case. Around 80% of these tumors are estrogen-secretes tumors, and the main symptom is irregular bleeding in the menstrual cycle or heavy menstrual bleeding as a consequence of endometrial hyperplasia (Szewczuk *et al*., 2020). Hence, the concomitant presence of endometrial cancer is observed in 5-25% of these patients (Abuali *et al*., 2022).

At the time of tumor diagnosis, our patient’s serum estrogen was in the normal reference interval (Figure 2). The presented patient’s initial symptom was just the opposite - the loss of spontaneous cycle and menstrual bleeding. The occurrence of secondary amenorrhea in these tumors can be explained by the suppression of FSH because of increased secretion of inhibin B and local inhibition of FSH by the increased paracrine activity of AMH (as evidenced by levels of serum hormones), which prevents normal follicle recruitment, ovulation, and regular menstruation (Li *et al*., 2018).

The present case of granulosa cell tumor with elevated LH and androgen levels without signs of hyperandrogenism or virilization is very rare (Ran *et al*., 2017). Pathophysiology of elevated LH levels is not completely clear. Still, the occurrence of hyperandrogenemia, anovulation, and secondary amenorrhea is a consequence of both high LH levels and local androgen activity on ovarian follicles (Gică *et al*., 2021). The malignant potential of granulosa tumors is low, and the stage of the disease at which treatment is initiated is the most significant prognostic factor for the patient. As about 90% of tumors are detected in stage I, survival is generally high at 86-96%. Treatment is surgical with additional chemotherapy for larger FIGO stages of the disease or because of iatrogenic dissemination of the disease (Ray-Coquard *et al*., 2018).

In this case, despite the patient’s reproductive age and desire to have a second child, chemotherapy was indicated due to iatrogenic dissemination of tumor tissue and finally larger stage of the disease. A sparing surgical approach to treatment and rejection of the recommended chemotherapy (which was solely the patient’s decision) helped restore the menstrual cycle and preserve fertility in the presented patient (Brink *et al*., 2022; Bergamini *et al*., 2019; Mangili *et al*., 2013). Spontaneous conception nine months after surgery, the normal course of pregnancy and the delivery of a second child, as well as another conception of a third pregnancy 36 months after surgery, all prove complete recovery of fertility previously lost due to granulosa cell tumor development (Guidi *et al*., 2021). Restoration of fertility in a short period after surgical treatment is a natural course of events and suggests that pregnancy and physiological amenorrhea that occur during pregnancy and postpartum can have a protective effect on the ovaries and prevent the possible recurrence of a malignant disease.

Chemotherapy indicated that iatrogenic dissemination of the tumor in a woman who wanted and planned to have children certainly poses a risk of a potential reproductive loss. Chemotherapy is not the only treatment option for women of reproductive age, as confirmed by our patient’s case (Brink *et al*., 2022; Bergamini *et al*., 2019). In addition to fertility preservation by gametes or embryo cryopreservation, drug-induced pseudopregnancy, amenorrhea, or even long-acting hormonal contraception could be treatment alternatives in some periods for patients who wanted to preserve fertility and were treated from ovarian tumors of low malignant potential (Dogan *et al*., 2022; Guidi *et al*., 2021; Zhang *et al*., 2017.)

## CONCLUSION

Complete restoration of fertility after a temporary loss due to granulosa cell tumor is possible after sparing surgical treatment. The postoperative natural course in woman of reproductive age, two spontaneous conceptions within 5 years after surgery, and the absence of disease recurrence despite the rejection of indicated chemotherapy (due to local iatrogenic dissemination of the disease) indicate a powerful protective role of pregnancy. In addition to fertility preservation by placing gametes, embryos, or sexual tissue in cryopreservation, induced pseudopregnancy, amenorrhea, or the use of long-acting hormonal contraception represent treatment options in patients who want to preserve fertility when there is a risk of fertility loss due to chemotherapy. Given the above, the presented case is in many ways interesting in clinical course, treatment, and recovery from the disease (Detti, 2021; Nejković *et al*., 2020; Wang *et al*., 2018; Iavazzo *et al*., 2015).
